# Construct validity of the Arabic version of the opinion relative to integration of student with disabilities scale

**DOI:** 10.1371/journal.pone.0343152

**Published:** 2026-02-13

**Authors:** Khalid N. Alasim, Bandar M. Almohayya

**Affiliations:** 1 College of Education, Special Education Department, Sattam bin Abdulaziz University, Al Kharj, Saudi Arabia; 2 College of Education, Special Education Department, University of Hail, Hail, Saudi Arabia; University of Hafr Al-Batin, SAUDI ARABIA

## Abstract

This study investigated the construct validity and psychometric properties of the Arabic version of the Opinion Relative to the Integration of Students with Disabilities (ORI) scale. Data were collected from 400 general and special education teachers as well as university faculty members. Exploratory factor analyses revealed a four-factor structure explaining 51.37% of the variance. Confirmatory factor analysis of the 15-item model demonstrated acceptable fit after item refinement. Subscale Cronbach’s α values ranged from.60 to.78, reflecting acceptable but marginal internal consistency in some subscales, with a total α of.75 (Cronbach’s α = .60–.78; total α = .75). Reliability indicators were further evaluated using Cronbach’s alpha and the convergent validity was assessed using the Average Variance Extracted (AVE), and Pearson correlation. The findings indicate that university faculty members and teachers generally hold positive attitudes toward inclusion and confirm that the Arabic ORI is a reliable and valid tool for assessing such attitudes. These results provide evidence supporting the instrument’s use in research and policy initiatives aimed at promoting inclusive education across Arabic-speaking contexts.

Diverse and complex attitudes exist toward individuals with disabilities [1,2]. Educators’ attitudes are among the most crucial factors influencing their roles and effectiveness in inclusive classrooms [3–7]. Al-Ahmadi [8] asserted that educators must have appropriately positive attitudes for inclusive classrooms to be successful. Positive attitudes among educators encourage the adoption of effective inclusive teaching practices and help address any unfavorable educational conditions within the classroom [9]. Further, favorable attitude toward inclusion contribute to greater engagement and communication between educators and students with disabilities [10]. According to D’Alonzo et al. [11], educators’ attitudes also influence their relationships with students and shape the attitudes of students without disabilities toward their peers with disabilities. Therefore, understanding educators’ attitudes toward inclusion and identifying ways to promote positive attitudes are critical for developing and implementing successful inclusion programs [7].

Teacher attitudes toward disability and inclusion can be explained through Ajzen’s Theory of Planned Behavior (1991) and Bandura’s Social Cognitive Theory (1986), which emphasize the role of beliefs, perceived norms, self-efficacy, and contextual factors in shaping behavior. Applied to inclusive education, these frameworks suggest that teachers’ readiness to implement inclusive practices is influenced by their beliefs, professional preparation, and surrounding environment. Although international initiatives such as the Salamanca Statement (1994) and the UNCRPD (2006) highlight the importance of teachers’ readiness for successful inclusion, attitudes toward inclusion continue to vary across cultural and educational contexts, including Arab countries such as Saudi Arabia.

Al-Abduljabber [12], Avramidis and Norwich [3], and Hung [13] revealed that the attitudes of educators, administrators, and students without disabilities significantly influence the education of students with disabilities in general education classrooms. Educators’ experiences in teaching and interacting with students with disabilities contribute to the development of their attitudes toward inclusion [10], while insufficient special education preparation and training remain a major factor shaping unfavorable attitudes [11]. Moreover, students with disabilities who encounter attitudinal barriers are less likely to participate in general education classroom and more likely to prefer separate settings. From the perspective of students with disabilities, teachers’ unfavorable attitudes represent one of the greatest obstacles to effective learning and positive peer interactions in inclusive classrooms [14,15].

Given this global and regional significance, it is essential to employ reliable and culturally appropriate tools to assess teachers’ attitudes toward inclusion. Educators’ attitudes have been measured using a variety of scales developed primarily in Western contexts to determine the factors that influence these attitudes [9,16]. For example, Cullen, Gregory, and Noto [17] developed the Teacher views Towards the Inclusion of Children with Disabilities into General Education Classrooms (TATIS). One potential drawback of this measure is that it omits certain crucial elements, such as classroom management and distinctions between special education and inclusive general education teachers. Another scale that has been used to measure educators’ attitudes toward the inclusion of students with learning or mild intellectual disabilities is the Regular Education Initiative Teacher Survey (REITS), which Semmel et al. [9] created. Many researchers have employed the REITS (e.g., [18]) to evaluate educators’ opinions regarding including students with mild disabilities alone. In addition, Cochran [19] created the Scale of Teachers’ Attitudes Towards Inclusive Classrooms (STATIC) to assess teachers’ attitudes toward including students with various types of disabilities.

However, these scales were developed and validated primarily in Western contexts and may not fully capture the linguistic, cultural, and educational nuances of Arabic-speaking countries. Direct application of English versions can lead to misinterpretation of items, reduced reliability, and inaccurate assessment of teachers’ attitudes.In particular, the Opinion Relative to the Integration of Students with Disabilities (ORI) scale, while psychometrically sound in English, has not undergone rigorous cross-cultural validation or confirmatory factor analysis in Arabic contexts. The ORI is a revised version of the Opinions Relative to Mainstreaming (ORM) developed by Larrivee and Cook [16] and later adapted by Antonak and Larrivee [2] based on construct validity and special education theories [20], consists of 25 items, in which 13 statements are worded positively on a six-point Likert scale that ranges from “Disagree very much” to “Agree very much” and 12 statements that are worded negatively. In addition, numerous terms from the original ORM were revised in the ORI to align more closely with contemporary special education terminology. Specifically, Antonak and Larrivee [2] changed the word “integration” to “mainstreaming,” and “child” and “children” to “student” and “students.” In addition, the authors chose “disabilities” for “handicapped” and “special need”.

Only exploratory factor analysis (EFA) was used to evaluate the ORI’s construct validity. As a parsimonious measure, Antonak and Larrivee [2] recruited a sample of 433 participants and employed orthogonal rotation methods. When the factor loading exceeded.37, the researchers attributed an item to a certain factor. Four factors were retained and the first, which accounted for 27% of the variance, had seven items (17, 14, 7, 3, 11, 21, 24, and 20) and was named *Benefits of Integration factor.* Ten items (18, 15, 12, 16, 4, 9, 25, 1, 6, 22) that were referred to as the *Integrated Classroom Management of Special Needs Children factor* constituted the second factor, which explained 7% of the variance. The third factor was responsible for 4% of the variance and had three items (9, 10, 2); it was referred to as *Perceived Ability to Teach Students with Disabilities.* The fourth factor accounted for 3% of the variance, had three items (5, 23, 13, 8), and was referred to as the *Special Versus Integrated General Education factor* [2]. These EFA results supported the construct validity of the revised ORI scale.

For the reliability indicators, the ORI’s Cronbach’s alpha coefficient was found to be.88, indicating high internal consistency [2]. Additionally, according to the Spearman-Brown reliability coefficient, the ORI’s split-half reliability was.82, and its standard error was 5.98. Nevertheless, neither the international versions nor the original English version underwent confirmatory factor analysis (CFA). Accordingly, the purpose of the present study was to examine the psychometric properties of the Arabic version of the ORI scale among teachers and university faculty members in Saudi Arabia. Specifically, this study aimed to evaluate the construct validity and internal consistency reliability of the Arabic ORI to determine its suitability for assessing attitudes toward the inclusion of students with disabilities within the Saudi educational context. To achieve these aims, the study addressed two central questions: 1) Does the Arabic version of the Opinions Relative to Integration of Students with Disabilities (ORI) scale demonstrate adequate construct validity? and 2) Does the Arabic version of the ORI scale demonstrate acceptable internal consistency reliability? This examination is important for understanding the measure’s validity and for assessing its structural and convergent validity. Since the revised English version of the original scale did not employ CFA to confirm the EFA findings, conducting CFA in this study was warranted.

## Methods

### Participants

The participants in the study were 102 faculty members from one Saudi public university and 298 special and general education teachers from 22 public elementary schools with self-contained classrooms for students with disabilities in Riyadh, Saudi Arabia. The selection of the sample of the study, for both teachers and university faculty, was made through theoretically informed sampling because of the role these groups play in shaping and driving the implementation of inclusive education practices at all levels in Saudi Arabia. The beliefs of classroom teachers determine whether inclusive educational policies and practices are successful or unsuccessful [21]. Furthermore, understanding university faculty attitudes toward inclusive education is crucial, as these attitudes serve as indicators of the success inclusion [22]. Therefore, teachers and faculty members represent the main stakeholders influencing inclusive practices.

The sample size for this study was also determined based on established psychometric guidelines to ensure adequate statistical power for factor analysis. Previous studies recommend that studies include 3–5 or up to 10 participants per questionnaire item to achieve stable factor extraction [23–25]. Given that the Arabic version of the ORI comprises 25 items, a minimum of 75–250 participants would be required according to these guidelines. The present study included 400 participants, exceeding these recommendations and thereby ensuring sufficient power and reliability for both exploratory and confirmatory factor analyses.

Table 1 shows the participants’ characteristics and demographics. The majority were men (56.5%), while 43.5% were women. Further, 46.6% of the sample were general education teachers, and 53.35% were special education teachers. All of the teachers together constituted 74.5% of the total sample, while faculty members representing 25.5%. A majority of the participating teachers held a bachelor’s degree (82.8%), while the remainder had postgraduate degrees. Regarding the participating faculty members’ educational level, the majority (98%) held higher degrees, while the remainder held a bachelor’s degree. Most teachers and faculty members had 1–10 years of experience, and the minority had more than 20 years of experience.

**Table 1:** The participants’ characteristics and demographics.

Variable	Level	N	%
Entire sample	Special and general education teachers	298	74.5
	Faculty members	102	25.5
Teachers	General education teachers	139	46.6
	Special education teachers	159	53.35
Teachers’ gender	Men	164	55
	Women	134	45
Level of education	Bachelor’s degree	247	82.8
	Post-graduate degree	51	17.1
Years of experience	One-ten years	122	40.9
	Eleven-twenty years	113	37.9
	Twenty-one to thirty-five years	63	21.1
Faculty members’ gender	Men	62	60.8
	Women	40	39.2
Educational level	Bachelor’s degree	4	4
	Graduate degree	98	96
Position	Teaching Assistant	6	5.9
	Lecturer	18	17.6
	Assistant Professor	57	55.9
	Associate Professor	15	14.7
	Full Professor	6	5.9
Years of experience	1-10 years	59	57.8
	More than 10 years	43	42.1
Country from which they obtained their last qualification	Saudi Arabia	35	34.3
	Arabic Countries	20	19.6
	United States of America	20	19.6
	United Kingdom	11	10.7
	Australia	2	1.9
	Others	14	13.7
Total Participants	400		

### Data collection

The researchers obtained permission to use the ORI from its author, Dr. Richard Antonak, and received ethical approval (No. SCBR-385/2024) from the Standing Committee for Bioethics Research (SCBR) at Prince Sattam bin Abdulaziz University. Written informed consent was obtained from all participants prior to data collection. Data collection was conducted from January 15, 2025, to March 10, 2025. For special and general education teachers, school visits were arranged to explain the study’s purpose and invite their participation. Teachers were encouraged to complete the scale within the designated period, and follow-up visits were conducted to remind and support participation. Faculty members were invited to participate via an electronic survey. The researchers initially sent the survey link through the university vice president for postgraduate studies and scientific research to the deans of colleges, who then circulated it to faculty members. Subsequently, the Deanship of Information Technology distributed the link via email to all faculty members. This electronic approach was selected due to the difficulty of reaching faculty members across all branch colleges.

### The Arabic version of the ORI

In the current study, the Arabic version of the ORI was utilized; therefore, the researchers, with the assistance of four multilingual subject matter experts, translated the original ORI from English to Arabic following the International Test Commission [26] Guidelines for Translating and Adapting Tests and the COSMIN standards. The forward–backward translation procedure was used to achieve both linguistic and conceptual equivalence for the instrument. The original English version was independently translated into Arabic by two bilingual experts and then back-translated into English by two other bilingual experts. Any discrepancies between the source version and the back-translated version were reviewed and consensus reached to ensure semantic accuracy and cultural relevance. Subsequently, content validity was evaluated by three experts in special education and psychometrics, who examined the Arabic version for clarity, relevance, and cultural appropriateness of each item. Upon completion, minor linguistic refinements were made based on their recommendations.

A pilot test of a large-scale administration was conducted using the translated Arabic version of the ORI. This pilot study included 38 teachers from the target population who were not included in the final sample. The objectives were to evaluate clarity, language accuracy and feasibility. The reliability analysis from the pilot study indicated a Cronbach’s alpha of 0.79, reflecting acceptable internal consistency. Based on participants’ feedback, items 3 and 13 were revised to improve clarity and readability.

The tool’s 25 items evaluated the educators’ views on including students with disabilities in general education programs on a 5-point Likert scale ranging from “Strongly Agree” to “Strongly Disagree.” Most Likert scales have a neutral anchor, which is crucial because it is anticipated that many respondents will feel neutral, although there is no set standard for the number of points. Accordingly, the five-point Likert scale used in this study helped reduce response bias [27]. A 5-point Likert scale is also adequate to assess reliability [28]. Moreover, numerous researchers have found that Likert scales between 2–7 points are similarly trustworthy [29].

### Data analysis

Listwise deletion was used to address any missing responses once the data was checked for missing values. Missing data accounted for approximately 2% of all responses. As the level of missing data was low, listwise deletion was considered appropriate and was applied across all analyses. Exploratory Factor Analysis (EFA) and Confirmatory Factor Analysis (CFA) were conducted using IBM SPSS Statistics version 25 and IBM SPSS AMOS version 25, respectively ([30,31]). The EFA served as the initial step in the data analysis. Factor analysis was used to assess the construct validity of the Arabic version of the ORI scale [32]. Cureton and D’Agostino [33] described factor analysis as “… a collection of procedures for analyzing the relations among a set of random variables observed or counted or measured for each individual of a group” (p. 1).

The suitability of applying factor analysis to the dataset was evaluated using the Kaiser-Meyer-Olkin (KMO) measure of sampling adequacy. A sample is considered adequate when KMO values fall between 0.8 and 1.0 [34]. The null hypothesis, according to the correlation matrix, was tested using Bartlett’s test of sphericity; for factor analysis to be appropriate, this test must yield a p-value less than.05 [35]. The varimax rotation method was employed to better understand the relationships among the items at a given level of factor analysis [36]. Factor extraction required a minimum factor loading > 0.4 and an Eigenvalue > 1.

Convergent validity and model fit of the measures were assessed using confirmatory factor analysis (CFA). Model fit was evaluated using the thresholds recommended by Hu and Bentler [37]: RMSEA ≤ 0.08, CFI ≥ 0.90, TLI ≥ 0.90, and SRMR ≤ 0.08. Cronbach’s alpha was used to examine internal consistency reliability of the scale and its subscales [38]. Several studies indicate that an alpha value of ≥.70 is adequate to assess an instrument’s reliability [39]. Cronbach’s alpha coefficients were interpreted as follows: ɑ ≥ .9 indicates exceptional reliability;.9 > ɑ ≥ .8, high reliability;.8 > ɑ ≥ .7, acceptable reliability;.7 > ɑ ≥ .6, doubtful reliability; and ɑ < .6, low reliability [40]. In addition, composite reliability (CR) was calculated to further assess internal consistency.

Following EFA, CFA was conducted using AMOS version 24.0 with maximum likelihood (ML) estimation. Model fit was evaluated using RMSEA, CFI, TLI, and SRMR, with thresholds based on Hu and Bentler [37].

## Results

The Arabic ORI’s mean and standard deviation were found to be (M = 2.90, *SD* = .48). This showed that the Approximation Rate to the Maximum Scores (ARMS) was moderate to high (58%), indicating that both the participating teachers and university faculty members demonstrated a generally positive attitude toward the integration of students with disabilities overall. Table 1 above shows the teachers’ and faculty members’ characteristics.

### Exploratory factor analysis

With a score of.87, the Kaiser-Meyer-Olkin (KMO) Measure of Sampling Adequacy was very good. In addition, the outcomes of Bartlett’s test of sphericity were significant. Given that both EFA assumptions were met, the analysis was appropriate for the data.

Principal axis factoring (PAF) was used to evaluate the Arabic version of the ORI scale’s validity after the data for this study were collected. Using promax rotation to rotate all five factors explained 54.67% of the overall variation.

As stated previously, just two loading items were found in one of the five rotational factors. In this case, many researchers recommend extracting a smaller number of factors [41–43]. Thus, to extract just four components, the researchers chose to run PAF once more. Four factors were rotated using promax rotation and the fixed factor approach and together they explained 51.37% of the total variance. The data were likely to factor well, as shown by the variables’ KMO Measure of Sampling Adequacy, which was.87. In addition, the total variation of the observed variables that each factor explained. Factor 1 explained 27% of the item’s variation, Factor 2, 10.72%, Factor 3, 7.27%, and Factor 4, 6.25%. In this fixed factor attempt, factor loadings were as follows: Factor 1: 7, 5, 3, 13, 24, 17, 14; 20 Factor 2: 12, 4, 18, 11, 6; Factor 3: 22, 16, 15, 10, 19, and Factor 4: 8, 9, 2. However, items 1, 23, 25, and 21 did not load as seen in Table 2.

**Table 2:** Factor loading and components of the Arabic version of ORI.

Item	Factor 1	Factor 2	Factor 3	Factor 4	*h2*
Item 7	.79				.69
Item 5	.78				.68
Item 3	.73				.56
Item 13	.67				.55
Item 24	.67				.54
Item 17	.65				.48
Item 14	.56				.58
Item 20	.49				.49
Item 12		.75			.60
Item 4		.70			.57
Item 18		.61			.53
Item 11		.56			.50
Item 6		.49			.36
Item 22			.73		.58
Item 16			.72		.60
Item 15			.63		.52
Item 10			.59		.55
Item 19			.53		.38
Item 8				.73	.57
Item 9				.66	.63
Item 2				.60	.42
Total *h2*					.56

Also, Table 3 presents the comparison of the factors in the English and Arabic versions of the scale, along with means and standard deviations for each item. In general, most items in the Arabic version loaded on the same factors as in the English version, indicating good conceptual equivalence between the two versions. More specifically, items 7, 5, 3, 24, 17, 14, and 20 loaded consistently under Factor 1 in both the original and Arabic versions. In addition, mean scores by items ranged between 2.98 and 3.89 (SDs = 1.01 to 1.34), reflecting general agreement and clarity of items across languages.

**Table 3:** Factor loading and components of the Arabic version of ORI versus English version.

Item	*Factor in the English Version*	Factor in the Arabic Version	Mean	*SD*
Item 7	Factor one	Factor one	3.17	1.34
Item 5	Factor one	Factor one	3.08	1.30
Item 3	Factor one	Factor one	3.89	1.01
Item 13	Factor four	Factor one	3.28	1.11
Item 24	Factor one	Factor one	3.07	1.22
Item 17	Factor one	Factor one	3.04	1.11
Item 14	Factor one	Factor one	3.33	1.11
Item 20	Factor one	Factor one	2.98	1.09
Item 12	Factor two	Factor two	3.14	1.14
Item 4	Factor two	Factor two	2.49	1.06
Item 18	Factor two	Factor two	3.25	1.14
Item 11	Factor one	Factor two	2.72	1.14
Item 6	Factor two	Factor two	2.40	1.08
Item 22	Factor two	Factor three	2.81	1.25
Item 16	Factor two	Factor three	2.87	1.30
Item 15	Factor two	Factor three	3.28	1.13
Item 10	Factor three	Factor three	2.71	1.12
Item 19	Factor three	Factor three	2.31	1.15
Item 8	Factor four	Factor four	2.21	1.07
Item 9	Factor two	Factor four	2.21	1.12
Item 2	Factor three	Factor four	1.55	.76

The following is an intuitive interpretation of the four factors. Most of the items entered into Factor 1 focus on the social and academic advantages of including students who have disabilities. Item 3, which states that inclusion provides diverse group interaction that promotes comprehension and acceptance of students’ differences, serves as an example. For instance, item 13 suggests that students with disabilities are likely to acquire academic skills more quickly in a regular classroom rather than a self-contained one. Items related to the conduct of students with disabilities in an inclusive classroom loaded onto Factor 2. For example, item 4 suggests that those students are likely to have behavioral issues in a regular classroom. The perceived capacity for teaching students with disabilities in inclusive classrooms was the subject of the third factor’s loaded items. Item 10, which stated that teachers in regular classrooms had the skills required to interact with students with disabilities, is one example. Finally, the items in Factor 4 dealt with the general classroom environment. For example, item 9 stated that giving children with disabilities more independence in a regular classroom causes too much confusion.

Further, item 14 has a component of the characteristic represented by Factor 2, although the PAF indicates that item 14 was associated most closely with Factor 1. In addition, none of the three items—1, 21, 23, and 25—loaded onto any factors.

### The Arabic version of the ORI’s reliability

According to Cronbach’s alpha, the reliability of all 25 items of the ORI employed in this study was.86. In particular, the value of each factor’s coefficient alpha is shown in Table 4, which presents the scale and the subscales’ reliability values.

**Table 4:** The Cronbach’s alpha values for the ORI; obtained with EFA.

Factor	No of items	Cronbach’s Alpha
Reliability overall	25	.86
Factor one	9	.86
Factor two	5	.73
Factor three	5	.72
Factor four	3	.60

### Confirmatory factor analysis

The measurement models that represent the hypothesized relations between indicators and factors were then assessed using CFA. Using 21 items from the first attempt derived from the EFA, the CFA revealed that the model did not fit the data well, as evidenced by the SRMR, RMSEA, CFI, and TLI values, which were.12 (SRMR > 0.08),.10 (RMSEA > 0.08),.68 (CFI < 0.90), and.64 (TLI < 0.90), respectively. Thus, the deletion technique was used as suggested by the modification indices; items 13, 14, and 18 were deleted and the second attempt was run using 18 items. The results of this second attempt improved the model slightly, but it was still inadequate. Its poor fit was indicated by the SRMR, RMSEA, CFI, and TLI values, which were.10 (SRMR > 0.08),.09 (RMSEA > 0.08),.78 (CFI < 0.90), and.74 (TLI < 0.90), respectively.

Next, based on the modification indices, items 7, 4, and 9 were deleted and the model was run again with 15 items. In this attempt, the model’s fit to the data was determined by the SRMR, RMSEA, CFI, and TLI values, which were.06 (SRMR ≤ 0.08),.08 (RMSEA ≤ 0.08),.88 (CFI < 0.90), and.85 (TLI < 0.90) for the four-factor structure. The model’s structure was deemed to be constructed appropriately, and the fitting optimization index was considered acceptable.

In addition, residual covariances were checked to improve the model by freeing error covariances between items from a single factor. For this, the values for SRMR, RMSEA, CFI, and TLI were.06 (SRMR ≤ 0.08),.05 (RMSEA ≤ 0.08),.92 (CFI ≥ 0.90), and.90 (TLI ≥ 0.90). Creating residual covariances required theoretical justification; covariances were added only for variables within the same factor based on the modification indices (items 5–20 and items 12–6). As seen in Table 5, therefore, the final 15-item four-factor solution provided an acceptable model fit according to Hu & Bentler’s [37] thresholds. Factor one included items 5, 3, 24, 17, 20; Factor two included items 12, 11, and 6; Factor three included items 22, 16, 15, 10, 19; and Factor four included items 8 and 2.

**Table 5:** Fit indices for all of the CFA’s attempts.

Fit Indices	*χ* ^*2*^	*(df)*	*(χ* ^*2*^ */df)*	TLI	CFI	RMSEA	GFI	NFI	AIC	SRMR	ECVI
Twenty-one items from EFA	1020.336	183	5.57	.64	.68	.10	.95	.64	23476.935	.12	2.88
Deletion of three items	572.512	129	4.43	.74	78	.90	.96	.74	20006.178	.10	1.72
Deletion of another 3 items	245.888	84	2.92	.85	.88	.06	.98	.83	16863.757	.06	.86
Residual covariance	198.598	82	2.42	.90	.92	.05	.98	.86	1682.46 7	.06	.75

Following the CFA, the ORI’s reliability was recalculated for the fifteen items and Cronbach’s alpha indicated that the scale’s scores overall were.70. In addition, each factor’s coefficient alpha was as follows, as shown in Table 6: Factor 1 (5 items) =.87, Factor 2 (3 items) =.70, Factor 3 (5 items) =.72, and Factor 4 (2 items) =.60. Factor 4’s Cronbach’s α of.60 indicates marginal internal consistency. While the other factors showed acceptable reliability. Composite reliability (CR) values for the 15-item Arabic ORI were also calculated to further assess internal consistency. The overall scale demonstrated a high level of reliability (CR = 0.92). Subscale CR values were as follows: Factor 1 = 0.80, Factor 2 = 0.63, Factor 3 = 0.78, and Factor 4 = 0.61. These results align with the Cronbach’s alpha findings and indicate generally acceptable reliability across the subscales, with somewhat lower reliability for Factor 2 and Factor 4, which include fewer items, which may reduce their measurement precision.

**Table 6:** The Cronbach’s alpha values for the ORI. N (15 items), produced by CFA.

Factor	No of Items	Cronbach’s Alpha
ORI Scale Overall	15	.70
Factor One	5	.78
Factor Two	3	.70
Factor Three	5	.72
Factor Four	2	.60

### Convergent validity

AVE values were computed for all four factors, and were required to establish that the factors’ convergent validities were ≥ .4. Although the composite reliability was higher than.6; (CR = 0.92), it was not the same for the factor two and factor four, as the AVE values were below the threshold level (.3). A Pearson Correlation was run next, and the values of the correlation between the subscales (factors) were positive and were all between.46 and.70; further, their correlations with the total factor was significant, which established the four factors’ convergent validity.

## Discussion

The purpose of this study was to use both the EFA and CFA to investigate the Arabic version of the ORI’s construct validity. Promax rotation was used to rotate five components. Although there were fewer components than those identified in Antonak and Larrivee’s [2] original study, this was expected because the instrument was translated into a different language and respondents from a different nation and culture provided the data. These five factors explained 54.67% of the variance. Further, one factor had only two loading items. Therefore, PAF was run again to extract four factors. The results showed that the KMO value for the four factors was.87, suggesting that the data were factored properly. More significantly, the four components combined explained 51.37% of the variance. In addition, it was found that Factor 1 accounted for 27.12% of the item variance, Factor 2 for 10.72%, Factor 3 for 7.27%, and Factor 4 for 6.25%. Although item 14 had aspects of the characteristic that Factor 2 described, it was most closely related to Factor 1. Items 1, 21, 23, and 25 did not load on any factor. Therefore, it is suggested that these items should be revised so that they load onto the proper variables for future study using the same scale. Consequently, this result shows that the four factors reflected the 25 items in partially, as the PAF was run two times to extract only four factors, instead of five factors.

The CFA’s results showed as well that the 25-items version of the scale had a poor factor solution. Thus, several attempts using deletion methods, modification indices, and residual covariance approaches showed that the four factors solution fit the data well with only 15 items. Further, most of the 15 items that were identified as the final set of items were found largely on its factor loading as Antonak and Larrivee [2] reported in their study (the only English psychometric analysis revision study conducted for this scale), in which the four-factor solution fit the data best. In addition, in this study, only items 19, 5, 2 loaded on different factors, while the other 12 items loaded on the same factors as in Antonak and Larrivee’s study.

Several of the items that were removed had weak or inconsistent factor loadings. It seems these are reflective of cultural and contextual issues in the meaning brought to certain items by respondents, rather than any problem with the constructs themselves. Some items focused on concepts that could be viewed differently within local educational contexts, increasing response variance and decreasing the strength of their relationships with the other items measuring the same factors. Eventually, items 24, 25, and 1 could not reach appropriate loadings and therefore were excluded from the final model. Such is the case with Item 24, showing inconsistent interpretations by the respondents, hence having a low loading on the supposed factor. Item 25 showed a poor loading pattern, likely due to conceptual ambiguity within the local educational context. Item one did not correlate statistically with related items; this implies that the item may have been perceived as addressing a different or less coherent construct by the respondents.

This reduction of the Arabic ORI from 25 to 15 items post-CFA was done to achieve a clear and psychometrically sound factor structure. The factor loadings for each retained item existed and made significant contributions to its sub-scale to yield a coherent four-factor model. The reliability analyses supported the robustness of the shortened scale, as the overall CR equaled 0.92, with the CR of the subscales ranging between 0.61 and 0.80; the latter two values for Factors 2 and 4 are somewhat low because of the smaller number of items forming these factors. The item reduction may have slight implications concerning content coverage; however, it could be said that the retained items adequately represent the core constructs, and the 15-item scale offers improved clarity, reduced participant burden, and practical usability. Further research could explore additional items within a theoretically grounded framework to further enrich specific subdomains while preserving the robust factor structure derived in this present study.

But this factor solution showed a good model fit across SRMR, RMSEA, CFI, and TLI values, all meeting the thresholds recommended by Hu and Bentler [37]. No study of the English version of the ORI using CFA was found in the literature. Our findings contribute significantly to the little research on cross-cultural validation in attitude scales such as the ORI. As far as we are aware, and because this was the first study to use the CFA to validate the EFA’s findings of the ORI scale, Therefore, comparison of the factorial analysis of the CFA is not possible.

The other comparison between Antonak and Larrivee’s [2] study and this study can include the factor content and meaning of the items within each factor. Therefore, we decided to incorporate covariances between items from the same factor. Because the two items derived from the same factor, we included residual covariances, and the covariance between the residuals for both items was reasonable. We made two attempts: the conceptual justification for Item 5 to Item 20’s was based on their derivation from Factor 1, and the conceptual justification for item 12 to item 6’s was based on their derivation from Factor 2, all of these pairs showed reasonable correlations between their residuals. The evaluation of the four elements revealed that the first two concentrate on 1) inclusion’s social and academic advantages, and 2) the behaviors of students with disabilities in inclusive classrooms. Each item’s content served as the theoretical basis for the use of the covariance between the residuals for both items in Factors 1 and 2. Moreover, before we added these residual covariances to the models, significant meaningfulness of each item content was considered, and the model was modified to produce one that fit better. These elements should be included in the model as they can make substantial substantive sense, particularly in social psychology research [44–46].

The validated Arabic version of the ORI scale provides a valuable tool for school and university administrators, researchers, and policymakers in Arabic-speaking contexts. For school and university administrators, it offers a standardized measure to assess and monitor teachers and faculty members’ attitudes toward inclusion, helping to identify professional development needs and to design targeted training programs that promote inclusive practices. Researchers can use this version to conduct cross-cultural studies, compare attitudes across regions or educational levels, and evaluate the impact of interventions aimed at improving inclusion. Policymakers may also benefit from using the scale as an evidence-based resource to guide inclusive education policies, allocate resources, and track progress toward national or regional goals in inclusive education. Overall, the availability of a psychometrically sound Arabic version of the ORI enhances opportunities for data-driven decision-making and supports the advancement of inclusive education across Arab countries.

For the reliability comparison, Antonak and Larrivee [2] recruited 433 participants, and the ORI’s Cronbach’s alpha coefficient was.88. This study recruited a slightly smaller number of participants (400), and the results indicated that the Cronbach’s alpha coefficients were acceptable for both the structures produced by the EFA and the CFA, although both tests incorporated significantly different sets of items; the EFA included 25 and the CFA 15 items only. However, it should be noted that Factor 4’s Cronbach’s α was.60, indicating marginal reliability. This suggests that some dimensions of the scale, particularly those with fewer items, may have limited power, and future studies could consider adding or refining items to improve the reliability of this subscale. The various participants in this study, which included university faculty members and general and special education teachers, might add more insight to the result of this study. For instance, this study recruited different faculty members who were Arab, but obtained their last qualifications from educational institutions in various countries. Those countries included Saudi Arabia, Arabic Countries, the United States of America, United Kingdom, Australia, and others. This is also similar in some ways to Antonak and Larrivee’s [2] study, in which they also recruited Arabs, African Americans, native Americans, and Hispanics in their study, as well as special and general education professionals. These similarities may add to the power of the results of both studies based upon the similarity in the participants’ characteristics, number, and nature.

### Future research and limitations

Future research should aim to extend the validation of the Arabic version of the ORI by testing its measurement invariance across different demographic and professional groups, such as gender, teaching experience, and educational level (e.g., elementary, middle, high school teachers, and faculty members). Establishing measurement invariance would provide stronger evidence that the scale measures the same construct equivalently across diverse populations. Additionally, researchers are encouraged to validate the scale with other stakeholder groups, such as parents of students with disabilities or students with disabilities themselves, to examine whether attitudes toward inclusion are similarly represented across different perspectives within the educational community. It would also be valuable to conduct longitudinal studies to track potential changes in attitudes over time following exposure to inclusive education policies or professional development initiatives. Finally, to enhance convergent and discriminant validity, future studies should include additional well-established measures that assess related constructs, such as teacher self-efficacy, inclusive teaching practices, and perceived institutional support for inclusion.

Despite its strengths, this study has several limitations that should be acknowledged. First, it relied on a self-report survey to collect data. Because such instruments depend on participants’ honesty and self-perceptions, responses may have been influenced by social desirability bias. Participants may have responded in ways they perceived as socially or professionally acceptable rather than fully reflecting their true attitudes, which could have led to slightly inflated scores on positive attitudes toward inclusion. Future research could address this limitation by incorporating anonymous surveys, using indirect measures of attitudes, including social desirability validity scales, or employing observational methods to complement self-report data. Second, the study’s sample was limited to elementary school teachers and university faculty members in Riyadh, which restricts the generalizability of the findings to educators in other regions of Saudi Arabia or other Arabic-speaking contexts.

A major limitation is the lack of discriminant validity analysis. Although convergent validity was assessed, the study did not formally evaluate whether the four factors of the Arabic ORI measure distinct constructs. This omission limits the strength of conclusions regarding the scale’s psychometric properties and should be addressed in future research, for example using the Fornell-Larcker criterion or HTMT ratio [47].

It should also be noted that combining teachers and university lecturers within the same analytical framework may introduce limitations related to measurement invariance, as these groups may differ in professional roles, experiences, and response patterns. Finally, much of the literature informing this study originated from Western contexts (e.g., the United States, the United Kingdom, and Australia), highlighting the need for more region-specific research on educators’ attitudes toward the inclusion of students with disabilities in Arab nations, particularly in Saudi Arabia.

## Supporting information

S1 Data (XLSX)
